# Occupational Heat Exposure and Chronic Venous Disease: Barriers, Adaptations, and Implications for Sustainable Workplaces

**DOI:** 10.3390/healthcare13233145

**Published:** 2025-12-02

**Authors:** Davide Costa, Michele Andreucci, Nicola Ielapi, Umberto Marcello Bracale, Raffaele Serra

**Affiliations:** 1 Interuniversity Center of Phlebolymphology (CIFL), International Research and Educational Program in Clinical and Experimental Biotechnology, University “Magna Graecia” of Catanzaro, 88100 Catanzaro, Italy; nicola.ielapi.research@gmail.com; 2 Department of Medical and Surgical Sciences, University “Magna Graecia” of Catanzaro, 88100 Catanzaro, Italy; andreucci@unicz.it; 3 Department of Public Health, University of Naples “Federico II”, 80131 Naples, Italy; umbertomarcello.bracale@unina.it

**Keywords:** chronic venous disease, heat exposure, sustainable workplaces, barriers, work environment

## Abstract

**Background:** Chronic venous disease (CVD) substantially influences workers’ comfort, productivity, and capacity to remain employed, yet many occupational settings complicate the implementation of effective symptom management strategies. Temperature fluctuations, in particular, influence daily functioning: heat often worsens swelling, heaviness, pain, and fatigue, while cold may offer partial relief. This study examines how workplace thermal and organizational conditions affect adults with CVD, paying attention to the challenges they face in applying clinical recommendations. **Methods:** Fifty adults with CEAP C1–C6 disease were interviewed and observed in clinical settings. A qualitative descriptive approach was adopted to capture workers’ accounts rather than generate a new theory. Data were analyzed using Braun and Clarke’s reflexive thematic analysis within a qualitative descriptive framework. **Results:** Heat exposure consistently aggravated swelling, pain, and fatigue, whereas moderate cold often improved comfort and functional capacity. Participants highlighted numerous workplace barriers, including rigid schedules, restrictive uniforms, and difficulties maintaining compression in hot environments. Supportive supervisors, micro-breaks, access to hydration, and flexibility in posture facilitated better symptom control. Workers frequently described tensions between clinical advice and job demands, noting that instructions such as leg elevation or frequent breaks were often unrealistic in their occupational context. **Conclusions:** Aligning clinical guidance with workplace realities is essential for the well-being and long-term employability of individuals with CVD. Climate-sensitive and ergonomic job design represents an important strategy for supporting employees’ ability to manage symptoms and sustain productivity amid increasing thermal variability.

## 1. Introduction

### 1.1. Chronic Venous Disease

Chronic venous disease (CVD) is a prevalent vascular condition affecting millions worldwide and has meaningful consequences for individual well-being, healthcare demand, and work performance [1,2]. Symptoms such as leg heaviness, swelling, pain, itching, and fatigue tend to fluctuate and are often influenced by environmental and occupational factors. Thermal conditions, in particular, appear to modulate symptom severity, with many patients reporting that heat worsens discomfort while cold alleviates it [1,2].

Although patient reports consistently highlight these temperature-related patterns, limited research has explored how environmental temperature affects venous symptoms in daily life or workplace settings. Most studies have concentrated on anatomical and physiological mechanisms, with relatively little attention given to workers’ experiences of managing symptoms across thermal conditions [3]. Recent climate extremes further underscore the significance of this issue. For example, the 2021 “Heat Dome” event in Canada produced wide-ranging occupational disruptions across 52 professions, demonstrating the vulnerability of workers exposed to high temperatures [4].

Advances in interventional and pharmacological treatment have improved clinical outcomes in CVD [1,2]. Nonetheless, conservative measures—including compression therapy, limb elevation, mobility breaks, and physical activity—remain essential components of symptom management [5]. Yet, in practice, many of these evidence-based recommendations are difficult to implement in workplace environments. Workers in kitchens, warehouses, construction sites, and office settings often encounter constraints that prevent consistent adherence to clinical advice [3,6,7].

From a broader perspective, sustainable workplace design has emerged as a determinant of employee well-being and organizational performance [8]. Sustainable workplaces not only consider environmental responsibility but also aim to create conditions that support long-term employee participation, especially among individuals with chronic diseases such as CVD. These approaches integrate ergonomic, psychosocial, and environmental considerations to optimize both health and productivity [9].

### 1.2. CVD: Pathophysiology and Clinical Implications

CVD arises from venous valve incompetence and venous hypertension, which contribute to edema, inflammation, skin changes, and ulceration [1,2]. The CEAP framework provides a standardized clinical classification (Table 1) [1,2,10]. Even early-stage disease can affect mobility and psychosocial well-being.

Thermal influences are an important but understudied factor in symptom fluctuation. Heat induces vasodilation, increases venous pooling, and intensifies swelling and discomfort, whereas cold produces vasoconstriction and may transiently reduce symptoms. However, excessive or prolonged cold exposure may also affect microcirculation [3,6,7,11]. Thus, individuals with CVD may face distinct challenges depending on temperature conditions.

Work capacity and participation can be further affected by the social visibility of symptoms and by workplace constraints that limit adherence to conservative management strategies. When clinical recommendations cannot be implemented, symptom severity may increase, potentially leading to functional decline.

### 1.3. Environmental Influences on CVD Symptoms and Challenges in Implementing Clinical Recommendations in Occupational Settings

Thermal environments strongly shape symptom patterns. Workers in hot environments—such as kitchens, factories, and outdoor labor—frequently report worsening swelling and fatigue, while individuals working in cold-storage facilities or cooler climates often describe temporary relief [3,6,7]. These fluctuations create daily challenges, especially when environmental conditions cannot be adjusted.

Clinical recommendations emphasize conservative interventions [12], yet adherence is often limited in real workplaces. Compression stockings may be uncomfortable in heat; mobility breaks conflict with rigid schedules; leg elevation is often impossible in public-facing or production-driven roles. Such mismatches illustrate ongoing gaps between guideline-based care and occupational feasibility [1,2,3,5].

Studies across different sectors reinforce this disconnect. Office workers often struggle to integrate mobility breaks; bakery workers experience intensified symptoms in elevated temperatures despite self-management attempts; and hospital food service staff face long periods of standing combined with substantial heat exposure [6,7]. These examples highlight structural barriers that make evidence-based care difficult to implement in many workplaces [13].

Individuals frequently develop informal coping strategies—discreet leg elevation, posture modification, or cooling the legs during breaks—to compensate for environmental constraints. Compression remains a foundational intervention, but adherence varies considerably depending on heat and comfort. Such strategies may provide short-term relief but are often insufficient without organizational support, particularly in fast-paced or inflexible environments [14,15,16,17].

Recent scholarship emphasizes the importance of sustainable lifestyle and workplace practices for both personal well-being and organizational performance [18,19,20,21,22,23]. Physical ergonomics, regular movement, and stress management contribute to CVD control, while rigid or unhealthy workplace environments may exacerbate symptoms. Sustainable employment requires designing systems that allow employees with chronic illnesses to work effectively without compromising health [23].

In practice, effective management of CVD depends not only on clinical recommendations but also on supportive workplace environments. Employers, occupational health professionals, and policymakers play key roles in enabling feasible symptom management through ergonomic seating, flexible scheduling, micro-breaks, and thermal accommodations [3,13].

From a broader societal perspective, integrating occupational health strategies with CVD management supports sustainability by promoting employee well-being, productivity, and economic resilience [24]. Supporting workers with chronic conditions is not simply a clinical responsibility—it is also a strategic investment in long-term organizational functioning [8,9].

By examining symptom fluctuations, adaptive strategies, and the alignment between clinical guidance and occupational realities, this study contributes to improving both scientific understanding and practical approaches to patient-centered care [1,2]. Specifically, it investigates how workplace thermal conditions affect the daily experience of CVD and how workers navigate constraints that limit adherence to medical recommendations.

## 2. Materials and Methods

### 2.1. Fieldwork and Participant Selection

This research adopted a qualitative descriptive design, chosen to capture the lived, embodied experiences of adults with CVD in their workplace context rather than to construct new theoretical models. This approach reflects a constructivist orientation, acknowledging that meaning is co-produced through interaction between participant and researcher. It was selected instead of phenomenological method because the primary aim was to describe barriers and adaptations that could inform workplace practice and policy.

Recruitment occurred at an outpatient vascular clinic. Inclusion criteria were age ≥ 18 years, clinical diagnosis of CVD (CEAP C1–C6), and current employment or employment within the past 12 months. Purposive sampling was used to ensure heterogeneity across occupational postures (standing, seated, mixed), thermal exposures (heat, cold, variable, climate-controlled), gender, and migration status. This variation enhanced transferability across sectors. Recruitment continued until thematic saturation—the point at which no new themes emerged.

A total of 50 participants were interviewed. Their socio-demographic and clinical features are summarized in Table 2.

### 2.2. Data Collection and Analytical Framework

Data collection combined semi-structured interviews and field observations. Interviews were conducted privately, lasted 45–75 min, and explored occupational tasks, thermal environments, symptom fluctuations, and the feasibility of clinical recommendations. Sample prompts included:

“How does temperature at work affect your legs?”

“What helps you manage discomfort during a shift?”

“Which pieces of medical advice are difficult to follow at work?”

Interviewers took field notes documenting nonverbal cues and contextual details within the clinical environment.

All interviews were conducted by two researchers with backgrounds in medical humanities and vascular medicine, respectively. Their positionality—one with clinical affiliation, one with qualitative ethnographic training—was acknowledged and discussed throughout analysis to reduce bias. Interviews were conducted in Italian, audio-recorded, transcribed verbatim, and translated into English. Translation was completed by a bilingual researcher and reviewed by the interviewers to ensure conceptual accuracy.

Reflexive thematic analysis, as outlined by Braun and Clarke [25], guided the analytic process. Transcripts were coded inductively, with initial codes developed by two researchers and reviewed during consensus meetings. A third researcher acted as an auditor, assisting in resolving discrepancies. Comparative matrices (e.g., CEAP classification × thermal exposure) supported cross-case analysis. Field observations informed theme refinement by providing contextual grounding. Member checking was not feasible due to logistical constraints; this limitation is acknowledged.

Table 3 presents the principal analytical themes and guiding questions.

### 2.3. Ethical Considerations

The study adhered to the Declaration of Helsinki and received ethical approval from the Institutional Review Board of the Interuniversity Center of Phlebolymphology (CIFL) International Research and Educational Program in Clinical and Experimental Biotechnology (ER.ALL.2018.75A). Written informed consent was obtained from all participants. Data were anonymized and potentially identifying workplace details were generalized. Reflexive audit trails were maintained.

## 3. Results

Thematic analysis identified four interrelated domains shaping how participants experienced and managed CVD at work:

1. Thermal strain and symptom fluctuations;
2. Workplace structures;
3. Adaptive self-management strategies;
4. Reconciling clinical advice with occupational realities;

These are summarized in Table 4.

### 3.1. Thermal Strain and Symptom Fluctuations

Participants consistently described temperature as a decisive influence on daily functioning. Heat—particularly in kitchens, factories, construction sites, and outdoor summer work—was associated with increased swelling, heaviness, pain, and fatigue. Many characterized heat as an “amplifier” that magnified baseline discomfort and, at times, interfered with concentration and job performance.

Conversely, cold environments such as refrigerated warehouses or outdoor winter work often produced noticeable relief. Participants emphasized that moderate cold reduced edema, provided temporary comfort, and improved endurance. Some described intentionally seeking cold exposure during breaks—cooling their legs with water or standing near cold air sources—to create brief periods of symptom relief. These accounts align with physiological findings that heat promotes vasodilation while moderate cold induces vasoconstriction.

At the same time, several participants noted that extreme or prolonged cold (e.g., unheated outdoor winter shifts) could trigger cramping or discomfort, suggesting a balance between beneficial and adverse effects.

*“On hot days, I feel my legs blow up like balloons. I move slower, I sweat more, and I get irritated with customers because the pain distracts me.”*

(Female, 39, retail worker)

*“In summer construction work, every hour feels double. The heat makes my veins throb, and even with compression, the pain doesn’t stop.”*

(Male, 51, construction worker)

*“When I was working in the cold storage room, my legs actually felt lighter. The swelling went down, and I could stand for longer without pain.”*

(Male, 55, warehouse worker)

*“I look forward to the winter season. In the cooler air, I feel more stable, almost like my veins can rest. It’s the only time I can wear compression without suffering.”*

(Female, 42, kitchen worker)

For many, cold environments provide an empowering sense of control over their condition, counterbalancing the unpredictability of symptom flare-ups in heat. Some participants even reported adopting informal strategies to recreate cold exposure during work, such as rinsing legs with cold water or sitting briefly near air conditioning units during breaks.

*“When the pain gets too much, I pour cold water on my legs in the bathroom. It gives me ten minutes of relief, which is enough to get through the shift.”*

(Female, 29, restaurant worker)

The accounts highlight a clear asymmetry between heat and cold: whereas heat intensified venous pooling through vasodilation, cold was described as protective, improving circulation and reducing symptom burden. Importantly, participants noted that these thermal fluctuations directly determined their daily endurance and capacity to remain productive. The unpredictability of symptoms across seasons and shifts meant that their ability to plan and sustain consistent self-management strategies was often undermined by temperature extremes.

*“I know my body now—if it’s over 30 degrees, I will suffer. If it’s under 15, I can work all day with half the pain. Temperature is like my real boss; it decides how the day goes.”*

(Male, 46, agricultural worker)

These lived accounts underscore that temperature is not a neutral background variable but an active determinant of disease burden, with profound implications for occupational health and sustainability. Heat exposure not only worsens physical symptoms but also diminishes concentration, morale, and overall job performance. Conversely, cold environments were consistently framed as a supportive condition, enabling workers to sustain productivity and maintain dignity in the workplace.

### 3.2. Workplace Structures That Exacerbate or Relieve Burden

Workplace organization strongly shaped symptom experiences. Rigid schedules, prolonged standing, limited opportunities to rest or change posture, and protective uniforms that trapped heat were frequently identified as aggravating factors. Participants described situations in which compression stockings became intolerable in high-heat environments, leading them to remove compression despite recognizing long-term consequences.

Supportive supervisors and flexible policies, however, substantially improved symptom management. Access to hydration stations, permission to take micro-breaks, and opportunities to sit briefly were described as simple but transformative accommodations. Participants framed these supports not only as physical relief but as gestures that increased their sense of dignity and recognition within the workplace.

*“We were not allowed to sit, even for a minute, outside official breaks. By closing time, my legs felt like they belonged to someone else.”*

(Female, 36, retail worker)

Heavy uniforms and protective clothing also intensified symptoms, particularly when combined with heat. Some participants described removing compression stockings or loosening garments despite knowing it would worsen swelling, as a way to survive the immediate discomfort.

*“The factory clothes are thick, and with stockings underneath, I can’t breathe. I know I’ll pay later, but sometimes I just have to take them off.”*

(Male, 44, factory worker)

Conversely, supportive environments were associated with strikingly different experiences. When supervisors allowed short pauses, hydration, or posture adjustments, participants reported feeling recognized and respected, which translated into both physical relief and psychological well-being.

*“My supervisor lets me sit for two minutes if I need to. It doesn’t slow the line, but it changes my whole day.”*

(Female, 41, assembly worker)

These contrasting accounts highlight that workplace structures are not neutral but active determinants of health. Rigid environments forced workers to abandon clinical recommendations, while supportive workplaces made symptom management feasible and sustainable. The difference was often described not in terms of medical outcomes alone, but in terms of dignity and fairness.

### 3.3. Adaptive Self-Management Strategies and Their Limits

Workers reported employing a range of self-management techniques—alternating posture, selective use of compression, applying cold packs, briefly elevating legs, or taking discreet movement breaks. These strategies offered some relief but were often constrained by job demands, pace of work, or stigma.

Compression was widely viewed as essential but difficult to tolerate during heat exposure. Cold packs melted quickly, and opportunities for leg elevation were limited in customer-facing roles. Some participants reduced visible adaptations to avoid being mistaken for “slow” or “unproductive.”

These narratives highlight the limits of individual effort when structural conditions remain inflexible.

*“The only thing that helps me is cold water. I put a bottle against my legs, and the relief is instant, even if it lasts only a short while.”*

(Female, 29, restaurant worker)

*“I sometimes lean against the freezer doors for a minute. It looks strange, but it cools my legs and gives me a break from the throbbing.”*

(Male, 52, supermarket worker)

However, participants also emphasized the limitations of these strategies. Compression stockings were considered essential but unbearable in hot climates; cold packs melted quickly in fast-paced roles; and discreet elevation of the legs was stigmatized or prohibited in customer-facing jobs. Some expressed frustration that their resilience and creativity were invisible to both employers and healthcare providers.

*“I try to stretch when the pain starts, but supervisors think I’m wasting time. So I stand still, even though it hurts more.”*

(Male, 51, construction worker)

These accounts reveal a paradox: while self-management was empowering in principle, it was rarely sustainable in practice. Workers often shouldered the responsibility of adaptation alone, even though its success depended on structural and cultural support. For many, the boundary between agency and resignation was thin, and the limits of self-management exposed the need for collective rather than individual solutions.

### 3.4. Reconciling Clinical Advice with Workplace Realities

Many participants expressed difficulty following clinical recommendations in their workplaces. Instructions to elevate legs, take regular breaks, or maintain continuous compression were viewed as unrealistic within fast-paced production environments or public-facing jobs [3,13]. Several workers concealed their diagnosis from employers due to fears of stigma, loss of shifts, or job insecurity. This concealment often prevented access to accommodations, underscoring the complexity of disclosure in precarious or hierarchical work contexts.

Overall, participants described a persistent gap between medical advice—which assumes autonomy, control over one’s schedule, and private space—and the realities of workplace structures, particularly in physically demanding or low-wage jobs.

*“The doctor tells me to put my legs up, but where? At the cashier desk? It’s impossible in my job.”*

(Female, 38, supermarket worker)

*“Wearing stockings in a 35 °C kitchen is unbearable. I want to follow the advice, but it feels like the doctor doesn’t understand what my work is like.”*

(Male, 43, chef)

Disclosure of the condition to employers was also fraught with difficulty. Several participants feared that revealing their illness would lead to stigma, fewer shifts, or even job loss. As a result, many concealed their symptoms, managing silently without accommodations.

*“I never told my boss about my condition. If they see me as weak, I’ll lose work. So I just stay quiet and suffer through it.”*

(Male, 45, gig worker)

These narratives underscore how medical advice often assumes autonomy, stability, and privacy—conditions not available in many workplaces, especially in low-wage or precarious jobs. The result was a structural inequality, where workers in flexible roles could adapt advice, while others were left unprotected. The negotiation between health and labor thus revealed not only individual struggles but also systemic gaps between healthcare and employment structures.

## 4. Discussion

This study provides evidence that workplace temperature and organizational conditions play a central role in shaping the daily experiences of adults with chronic venous disease [3,26]. Heat consistently aggravated symptoms, while moderate cold often offered relief, reinforcing known physiological mechanisms linking thermal exposure to venous function [27,28,29].

### 4.1. Environmental Conditions as Determinants of Venous Health

Thermal exposure acts as an occupational hazard that directly influences work capacity. The patterns described by participants align with the vasodilatory effects of heat and the vasoconstrictive effects of cold [3,6,7,11].

At the same time, the results extend existing literature by situating these mechanisms within real occupational settings [7]. Workers’ accounts reveal how environmental, organizational, and social factors interact to determine whether clinical recommendations are feasible. Such insights emphasize that symptom management cannot be isolated from working conditions or employment policies.

The findings also align with recent research on occupational climate stress. During Canada’s 2021 “Heat Dome,” Tetzlaff et al. [4] reported extensive heat-related disruptions across 52 professions, highlighting temperature as a determinant of work safety and continuity. Our participants’ testimonies reflect the same vulnerability—particularly among those with pre-existing vascular disease—underscoring the need for integrated climate-adaptation planning in labor policy.

From a methodological standpoint, using Braun and Clarke’s reflexive thematic analysis [25] enabled transparent coding and theme generation, supported by iterative team dialog and reflexive memos.

### 4.2. Organizational Flexibility and Managerial Support

Supervisory attitudes and workplace policies emerged as decisive factors in enabling symptom management. Accommodations such as micro-breaks, hydration access, ventilation, and posture adjustments align with broader evidence linking organizational culture to health and productivity [8,9,30,31]. These interventions are relatively low-cost yet offer significant benefits.

### 4.3. Constraints on Self-Management

Participants demonstrated creativity in adapting to thermal discomfort and occupational constraints. Yet self-management alone was seldom adequate. Without structural support, workers were often forced to choose between short-term comfort and long-term clinical needs. This imbalance places disproportionate responsibility on individuals and may exacerbate inequities [32,33].

### 4.4. Interactions Between Thermal Exposure, Chronic Disease, and Inequality

Important patterns emerged regarding thermal stress and unequal workplace vulnerability. Migrant and precarious workers—overrepresented in physically demanding and heat-exposed roles—reported fewer accommodations, less flexibility, and greater stigma [34,35,36,37,38,39]. Women described unique challenges related to uniforms, social expectations, and visibility of symptoms. These findings parallel broader evidence of intersectional inequalities in occupational health [39,40,41].

### 4.5. Clinical–Occupational Misalignment

Clinical guidelines emphasize approaches that presuppose control over scheduling, mobility, and environment—these conditions are often absent in fast-paced or low-autonomy jobs [3,11,34,35]. Participants’ accounts reflect a structural disconnect that may contribute to disengagement from medical advice and reinforce stigma [36,37,38]. Strengthening communication between clinicians, workers, and employers may help produce more feasible, individualized care plans [42,43].

### 4.6. Practical Implications for Workplace Adaptation

Findings support several actionable strategies:

(1) Engineering controls: ventilation, localized cooling, redesign of heat-retaining uniforms.

(2) Administrative adjustments: flexible break schedules, task rotation, micro-breaks, temperature zoning.

(3) Clinical collaboration: clinicians asking targeted questions about work conditions, providing occupation-specific guidance.

(4) Equity measures: prioritizing migrant and precarious workers who face disproportionate exposure to heat.

### 4.7. Cold Exposure: Nuance and Caution

While cold was often described as beneficial, prolonged exposure may impair microcirculation in severe disease. Only two participants had advanced C5–C6 disease, limiting generalizability. Future research should assess optimal temperature ranges and durations for safe symptom relief.

### 4.8. Methodological Strengths and Limitations

Strengths include sample diversity, methodological rigor, and reflexive analysis. Limitations include:

(1) The reliance on self-reported data introduces potential recall bias.
(2) Participants were recruited from a single Italian region and clinical setting, which may limit generalizability.
(3) Objective thermal or physiological measures (e.g., temperature logs, edema metrics) were not collected.
(4) Individuals who left employment due to CVD may be under-represented.

Nevertheless, sample diversity and thematic saturation support analytic robustness, and the reflexive approach provided transparency regarding researcher influence and interpretation.

## 5. Conclusions

Workplace temperature and organizational conditions profoundly shape the daily experiences of individuals with chronic venous disease. Heat exacerbates symptoms, while moderate cold may provide relief. Organizational rigidity, restrictive uniforms, and stigma intensify the burden, whereas supportive supervision, micro-breaks, and access to hydration enhance workers’ capacity to adhere to clinical advice.

From a sustainability perspective, integrating chronic disease management into workplace design is essential. Aligning clinical guidance with occupational realities supports long-term workforce participation, equity, and organizational resilience as thermal variability increases and populations age.

## Figures and Tables

**Table 1:** CEAP Clinical (C) Classification.

C0: No visible or palpable signs of venous disease.
C1: Telangiectasias (spider veins) or reticular veins.
C2: Varicose veins.
C2r: Recurrent varicose veins.
C3: Edema (swelling).
C4: Skin changes due to chronic venous disease.
C4a: Pigmentation and/or eczema.
C4b: Lipodermatosclerosis or atrophie blanche.
C4c: Corona phlebectatica (a web of fine, blue-violet, and superficial veins).
C5: Skin changes (as in C4) with healed venous ulceration.
C6: Skin changes (as in C4) with active venous ulceration.
C6r: Recurrent venous ulceration.

**Table 2:** Characteristics of participants.

Variable	Category	*n*	%
Age group	18–39	12	24%
	40–59	22	44%
	60+	16	32%
Gender	Female	34	68%
	Male	16	32%
Job posture	Primarily standing	21	42%
	Primarily seated	15	30%
	Mixed	14	28%
Thermal exposure	Heat-exposed	14	28%
	Cold-exposed	8	16%
	Thermally variable	15	30%
	Climate-controlled	13	26%
Disease severity (CEAP)	C1	8	16%
	C2	24	48%
	C3	10	20%
	C4	6	12%
	C5	1	2%
	C6	1	2%
Migration status	Native-born	33	66%
	Migrant	17	34%
Employment type	Full-time	31	62%
	Part-time/seasonal/gig work	19	38%

**Table 3:** Key analytical themes and guiding questions.

Theme	Subthemes/Focus	Guiding Questions
Thermal strain and symptom fluctuations	Heat-related discomfort; cold-related pain; variability	How do workers describe temperature effects on symptoms and daily functioning?
Workplace structures	Tasks; schedules; PPE/uniforms; access to cooling/heating	Which organizational or physical factors exacerbate or relieve burden?
Self-management strategies	Compression; breaks; cooling/heating improvisation	What strategies do workers adopt, and how effective are they?
Negotiating clinical advice	Adherence barriers; workplace mismatch; stigma	How do patients reconcile clinical advice with job realities?

**Table 4:** Thematic prevalence and illustrative participant experiences (*n* = 50).

Theme	% of Participants Mentioning Theme	Illustrative Experiences
Thermal strain and symptom fluctuations	80%	Workers reported swelling and heaviness in hot kitchens and outdoor summer tasks; cramps and pain in refrigerated warehouses and outdoor winter jobs. Symptoms varied with rapid temperature changes.
Workplace structures	70%	Rigid schedules and heavy protective uniforms increased discomfort. Access to hydration, micro-breaks, and supportive supervisors reduced symptom burden.
Self-management strategies	64%	Patients improvised with compression wear, cooling packs, posture changes, and covert breaks. Many described limits: stockings unbearable in heat, cooling packs impractical in fast-paced work.
Negotiating clinical advice vs. workplace realities	58%	Advice to elevate legs or take regular breaks was seen as unrealistic. Disclosure to employers was fraught with stigma and fear of job insecurity. Some adapted advice discreetly.

## Data Availability

The raw data supporting the conclusions of this article will be made available by the authors on request.
